# Association Between Triglyceride-Glucose Index and Hypertension: A Meta-Analysis

**DOI:** 10.3389/fcvm.2021.644035

**Published:** 2021-05-31

**Authors:** Yi Wang, Wei Yang, Xiao Jiang

**Affiliations:** ^1^Department of Outpatient, The First Affiliated Hospital of Jinan University, Guangzhou, China; ^2^Department of Internal Medicine, Guangdong Women and Children Hospital, Guangzhou, China; ^3^Department of Plastic Surgery, The First Affiliated Hospital of Jinan University, Guangzhou, China

**Keywords:** triglyceride-glucose index, hypertension, insulin resistance, observational studies, meta-analysis

## Abstract

**Background:** Triglyceride-glucose (TyG) index is a recently proposed surrogate indicator of insulin resistance. Previous studies evaluating the association between TyG index and hypertension risk in general adult population showed inconsistent results. We performed a meta-analysis to systematically evaluate this association.

**Methods:** Observational studies, which evaluated the independent association between TyG index and hypertension in the general adult population, were identified by systematic search of PubMed, Embase, Web of Science, Wanfang data, and Chinese National Knowledge Infrastructure databases. A random-effect model, which incorporated the potential intra-study heterogeneity, was used for the meta-analysis.

**Results:** Eight observational studies including 200,044 participants were included. Results showed that compared with those with the lowest category of TyG index, subjects with the highest category of TyG index were associated with higher odds of hypertension [adjusted risk ratio (RR): 1.53, 95% confidence interval (CI): 1.26–1.85, *I*^2^ = 54%, *P* < 0.001]. Sensitivity analysis by excluding one dataset at a time showed consistent result (adjusted RR: 1.44–1.62, *P* all < 0.001). Results of univariate meta-regression analysis showed that differences in sample size, mean age, male proportion, mean body mass index, and study quality score among the included studies did not have significant influence on the association between TyG index and hypertension (*P* values all > 0.10), suggesting that differences in these characteristics may not be the major source of heterogeneity. Subgroup analyses showed that study characteristics such as study design, participant ethnicity, age, or sex of the participants did not significantly affect the association (*P* for subgroup difference all >0.05).

**Conclusions:** Higher TyG index may be associated with higher odds of hypertension in general adult population. Large-scale prospective cohort studies are needed to validate these findings, and further studies are needed to elucidate the potential pathophysiological mechanisms underlying the association between TyG index and hypertension.

## Introduction

Currently, hypertension remains an important cause of morbidity and mortality of global population (1), particularly for people from the developing countries (2). Early identification of population at higher risk for the development of hypertension is critical to reduce the incidence of the disease and its cardiovascular complications (3). Previous studies showed that insulin resistance may be involved in the pathogenesis of hypertension via mediating low-degree systematic inflammation (4, 5). Conventionally, hyperinsulinemic–euglycemic clamp test is considered as the “gold standard” method for the evaluation of insulin sensitivity (6). However, application of hyperinsulinemic–euglycemic clamp test in real world clinical practice is limited since this method is time consuming and expensive (7). Recently, triglyceride-glucose (TyG) index, a fasting blood glucose and triglyceride synthesis parameter, has been proposed as a reliable indicator of insulin resistance (8). The TyG index is a non-insulin-based index that is inexpensive and could be easily obtained based on a single sample, which has been suggested as a reliable surrogate biochemical marker of insulin resistance (9). Accumulating evidence showed that higher TyG index is independently associated with and increased risk of type 2 diabetes mellitus in general population (10). However, previous studies evaluating the association between TyG and hypertension risk in community-derived adult population showed inconsistent results (11–18). Therefore, in this study, we aimed to evaluate the association between TyG index and hypertension in general adult population via a meta-analysis of observational studies. Moreover, potential influences of characteristics of the participants on the association were also analyzed.

## Methods

The meta-analysis was performed in accordance with the MOOSE (Meta-analysis of Observational Studies in Epidemiology) (19) and Cochrane's Handbook (20) guidelines.

### Literature Search

Studies were identified via systematic search of electronic databases of PubMed, Embase, Web of Science, Wanfang data, and Chinese National Knowledge Infrastructure (CNKI) databases via the following terms: (1) “TyG index” OR “triglyceride-glucose index” OR “triglyceride and glucose index”; and (2) “hypertension” OR “blood pressure” OR “hypertensive.” The search was limited to human studies published in English or Chinese. The reference lists of related original and review articles were also analyzed using a manual approach. The final literature search was performed on March 10, 2021.

### Study Selection

The inclusion criteria for the studies were: (1) observational studies published as full-length articles; (2) included general adult population; (3) evaluated the association between TyG index and hypertension; and (4) reported the relative risk for this association after adjustment of potential confounding factors. TyG index was calculated as ln [TG (mg/dl) × FPG (mg/dl)/2] (21). Diagnosis of hypertension was in accordance with the criteria applied in the included studies, which was generally defined as systolic BP ≥ 140 mmHg, diastolic BP ≥ 90 mmHg, or on treatment of antihypertensive medications. Reviews, editorials, preclinical studies, and studies irrelevant to the aim of current meta-analysis were excluded. Besides, studies including participants <18 years old, focusing on patients with confirmed diagnosis of certain diseases rather than general population, without measuring of TyG index, or that reported data based on univariate analyses rather than multivariate analyses were excluded from the meta-analysis.

### Data Extracting and Quality Evaluation

Literature search, data extraction, and quality assessment of the included studies were performed by two authors (YW and WY) independently according to the predefined inclusion criteria. Discrepancies were resolved by consensus. The extracted data included: (1) name of first author, publication year, and country where the study was performed; (2) study design characteristics; (3) participant characteristics, including health status, sample size, age, sex, and body mass index (BMI); (4) patterns for Lp (a) analysis and cutoff values; (5) follow-up durations for cohort studies; (6) definitions of hypertension and methods for outcome validation; and (7) confounding factors adjusted in the multivariate analyses. The quality of each study was evaluated using the Newcastle-Ottawa Scale (22), which ranges from 1 to 9 stars and judges each study regarding three aspects: selection of the study groups; the comparability of the groups; and the ascertainment of the outcome of interest.

### Statistical Analyses

We used risk ratios (RRs) and their corresponding 95% confidence intervals (CIs) as the general measure for the association between TyG index and hypertension in general adult population. For all of the included studies, TyG index was analyzed as categorized variables. Accordingly, RRs of hypertension in adults with the highest TyG index level compared with those with the lowest TyG index level were extracted. Data of RRs and their corresponding stand errors (SEs) were calculated from 95% CIs or *P* values, and were logarithmically transformed to stabilize variance and normalize the distribution (20). The Cochrane's *Q* test and estimation of *I*^2^ statistic were used to evaluate the heterogeneity among the included cohort studies (23). A significant heterogeneity was considered if *I*^2^ > 50%. We used a random-effect model to synthesize the OR data because this model is considered as a more generalized method, which incorporates the potential heterogeneity among the included studies (20). Sensitivity analyses, by omitting one individual study at a time, were performed to test the robustness of the results (24). A univariate meta-regression analysis was performed to evaluate the potential influences of study characteristics including sample size, mean age, male proportion, mean BMI, and study quality score on the association between TyG index and odds of hypertension (20). Besides, predefined subgroup analyses were performed to evaluate the influences of study characteristics on the outcome, including study design, ethnicity, age, and sex of the participants. Briefly, median of continuous value was chosen as cut-off value, and studies were grouped according to the study design (cohort or cross-sectional studies), ethnicity of the participants (Chinese or non-Chinese), mean age (< or ≥50 years), and sex of the subjects (male or female). Subsequent comparisons for the outcome within subgroups were performed with Chi-square test. The potential publication bias was assessed by visual inspection of the symmetry of the funnel plots, as well as the Egger's regression asymmetry test (25) and Begg's test (20). A *P* < 0.05 was considered as statistically significant. We used the RevMan (Version 5.1; Cochrane Collaboration, Oxford, UK) and STATA software for the meta-analysis and statistics.

## Results

### Literature Search

**Figure 1:**
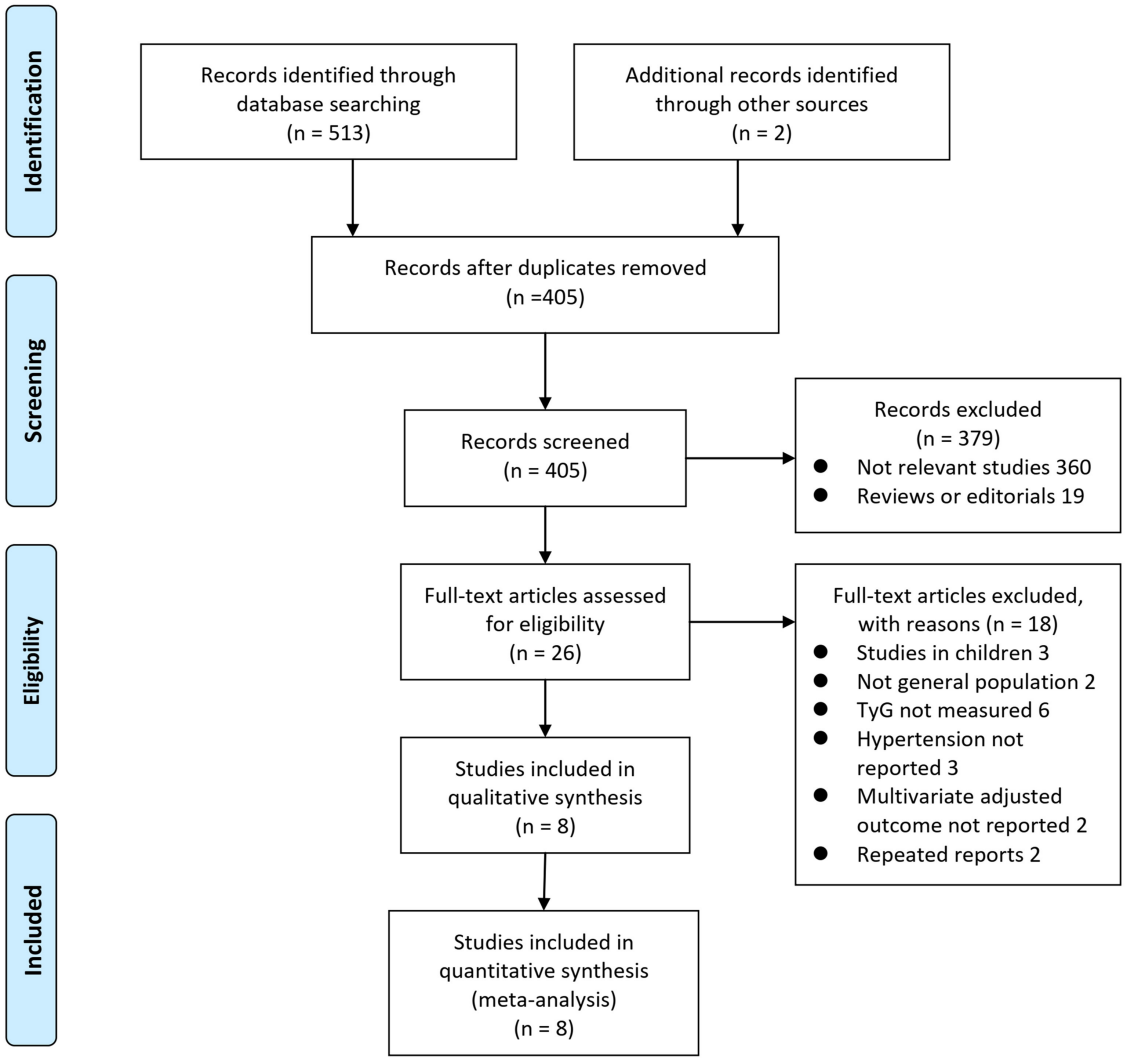
Flowchart of database search and study identification.

The process of database search is summarized in Figure 1. Briefly, 405 articles were found via initial literature search of the databases after excluding of the duplications. Among them, 379 were excluded through screening of the titles and abstracts mainly because they were not relevant to the purpose of the meta-analysis. Subsequently, 26 potential relevant records underwent full-text review. Of these, 10 were further excluded for the reasons listed in Figure 1. Finally, eight observational studies were obtained for the meta-analysis (11–18).

### Study Characteristics and Quality Evaluation

The characteristics of the included studies are summarized in Table 1. Overall, eight with 200,044 adult participants from community population were included. The studies were performed in Spain (11), Romania (14), Mexico (16), and China (12, 13, 15, 17, 18). Regarding study design, two of them were prospective cohort studies (11, 13), and the remaining six were cross-sectional studies (12, 14–18). The sample size of the included studies varied between 542 and 142,005. The mean ages of the participants among each study ranged from 39 to 61 years, and the mean BMI varied from 22.7 to 27.2 kg/m^2^. For the analysis of the association between TyG index and odds of hypertension, comparisons were performed between participants with highest and lowest quintiles in two studies (11, 18), between those with highest and lowest quartile in five studies (12–15, 17), and according to a cutoff value of 4.68 in one study (16). Validation of hypertension outcome was performed by clinical examination by trained research members. Age, sex, BMI, smoking status, and other potential confounding factors were generally adjusted to a varying degree when the association between TyG index and hypertension was reported. The NOS scores of the included studies ranged from eight to nine, indicating generally good study quality.

**Table 1:** Characteristics of the included observational studies.

**Study**	**Country**	**Design**	**Participant characteristics**	**Sample size**	**Age**	**Male**	**BMI**	**TyG analysis**	**Follow-up duration**	**Diagnosis of hypertension**	**Outcome validation**	**Variables adjusted**	**NOS**
					**Years**	**%**	**kg/m^**2**^**						
Sanchez-Inigo et al. (11)	Spain	PC	General population without hypertension at baseline	3,637	51.9	60.2	26.4	Categorized (Q5:Q1)	8.5	SBP ≥ 140 mmHg, DBP ≥ 90 mmHg, or initiation of antihypertensives	Clinical examination by trained physicians	Age, sex, BMI, smoking, alcohol intake, lifestyle pattern, T2DM, LDL-C, SBP, DBP, and antiplatelet therapy	9
Jian et al. (12)	China	CS	Community population	1,777	60.8	42.1	24.8	Categorized (Q4:Q1)	NA	SBP ≥ 140 mmHg, DBP ≥ 90 mmHg, or on antihypertensives	Clinical examination by trained members	Age, sex, BMI, WHR, smoking, family history of hypertension, educational level, marital status, and family income	9
Zheng and Mao (13)	China	PC	General population without hypertension at baseline	4,686	40.5	67.8	23.1	Categorized (Q4:Q1)	9	SBP ≥ 140 mmHg, DBP ≥ 90 mmHg, or on antihypertensives	Clinical examination by trained members	Age, sex, BMI, WC, BUN, SCr, FPG, UA, AST, ALT, γ-GGT, TC, TG, HDL-C, LDL-C, Apo-A1, Apo-B, and eGFR	9
Liu et al. (15)	China	CS	General population not on antihypertensives	142.005	43.7	58.6	23.9	Categorized (Q4:Q1)	NA	SBP ≥ 140 mmHg, or DBP ≥ 90 mmHg,	Clinical examination by trained examiners	Age, sex, and smoking	8
Bala et al. (14)	Romania	CS	Community population	1,730	46.8	46.7	27.2	Categorized (Q4:Q1)	NA	SBP ≥ 140 mmHg, DBP ≥ 90 mmHg, or on antihypertensives	Clinical examination by trained members	Age, sex, smoking, drinking, sedentary lifestyle, eGFR, urinary sodium, and UACR	9
Zhu et al. (18)	China	CS	General population aged over 40 years	43,591	56.8	29.6	24.2	Categorized (Q5:Q1)	NA	SBP ≥ 140 mmHg, DBP ≥ 90 mmHg, or on antihypertensives	Clinical examination by trained clinicians	Age, center, sex, history of CVDs, history of T2DM, hypoglycemic drugs, SBP, DBP, BMI, ALT, AST, WHR, eGFR, smoking, and drinking	9
Morales-Gurrola et al. (16)	Mexico	CS	Community population	542	39.3	32.7	22.7	Categorized, cutoff: 4.68	NA	SBP ≥ 140 mmHg, DBP ≥ 90 mmHg, or on antihypertensives	Clinical examination by trained examiners	Age, sex, BMI, and WC	8
Wang et al. (17)	China	CS	Community population	2,076	58.6	39.9	25.1	Categorized (Q4:Q1)	NA	SBP ≥ 140 mmHg, DBP ≥ 90 mmHg, or on antihypertensives	Clinical examination by trained members	Age, sex, BMI, family history of hypertension, smoking, alcohol drinking, TC, HDL-C, and LDL-C	9

*BMI, body mass index; TyG, triglyceride–glucose index; NOS, Newcastle-Ottawa Scale; PC, prospective cohort; CS, cross-sectional; Q5:Q1, the 5th quintile vs. the 1st quintile; Q4:Q1, the 4th quartile vs. the 1st quartile; NA, not applicable; SBP, systolic blood pressure; DBP, diastolic blood pressure; T2DM, type 2 diabetes mellitus; LDL-C, low-density lipoprotein cholesterol; WHR, waist-to-hip ratio; WC, waist circumference; BUN, blood urea nitrogen; SCr, serum creatinine; FPG, fasting plasma glucose; UA, uric acid; ALT, alanine aminotransferase; AST, aspartate aminotransferase; γ-GGT, γ-glutamyl transpeptadase; TC, total cholesterol; TG, triglyceride; HDL-C, high-density lipoprotein cholesterol; eGFR, estimated glomerular filtrating rate; UACR, urinary albumin creatinine ratio; CVDs, cardiovascular diseases*.

### Association Between TyG Index and Hypertension

**Figure 2:**
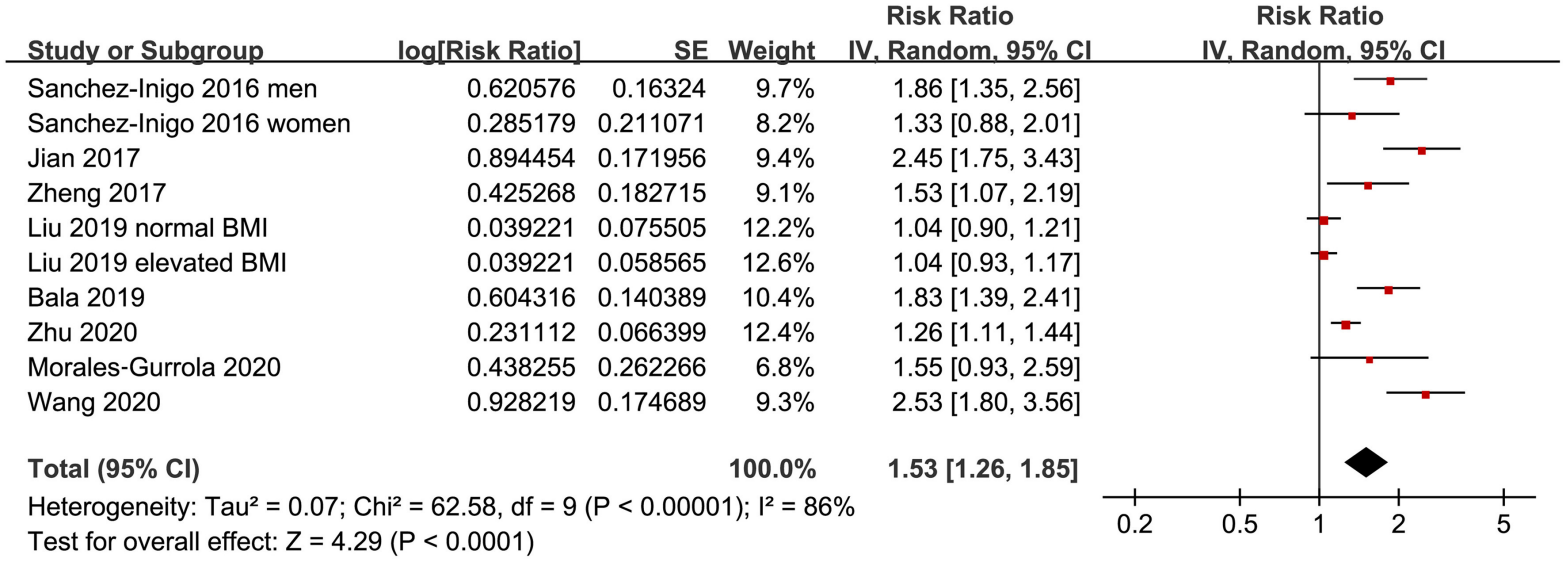
Forest plots for the meta-analysis of the association between triglyceride-glucose (TyG) index and hypertension in community-derived adult population.

**Figure 3:**
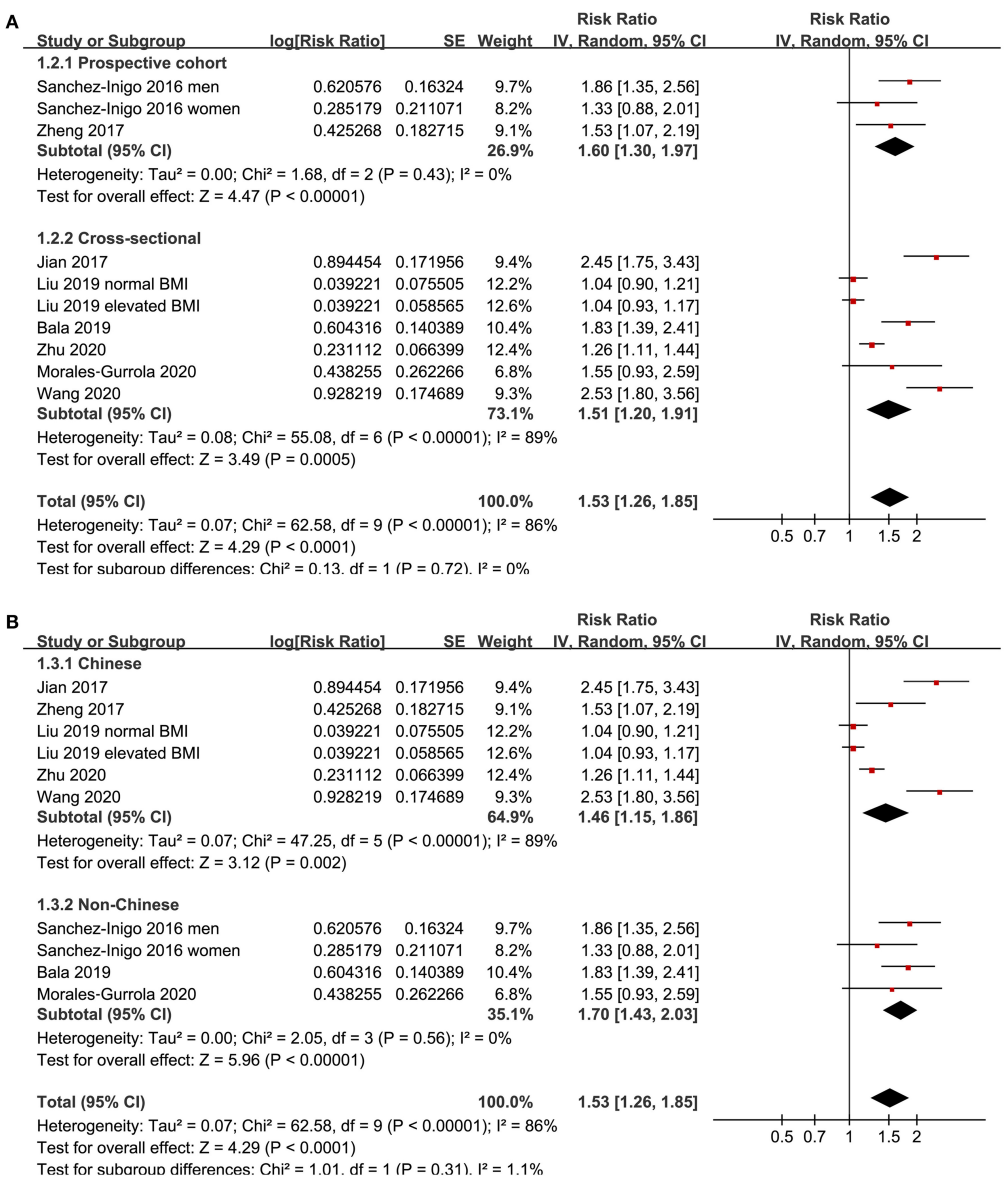
Subgroup analyses for the association between TyG index and hypertension in community-derived adult population; **(A)** subgroup analysis according to the study design; and **(B)** subgroup analysis according to the ethnicity of the population.

**Figure 4:**
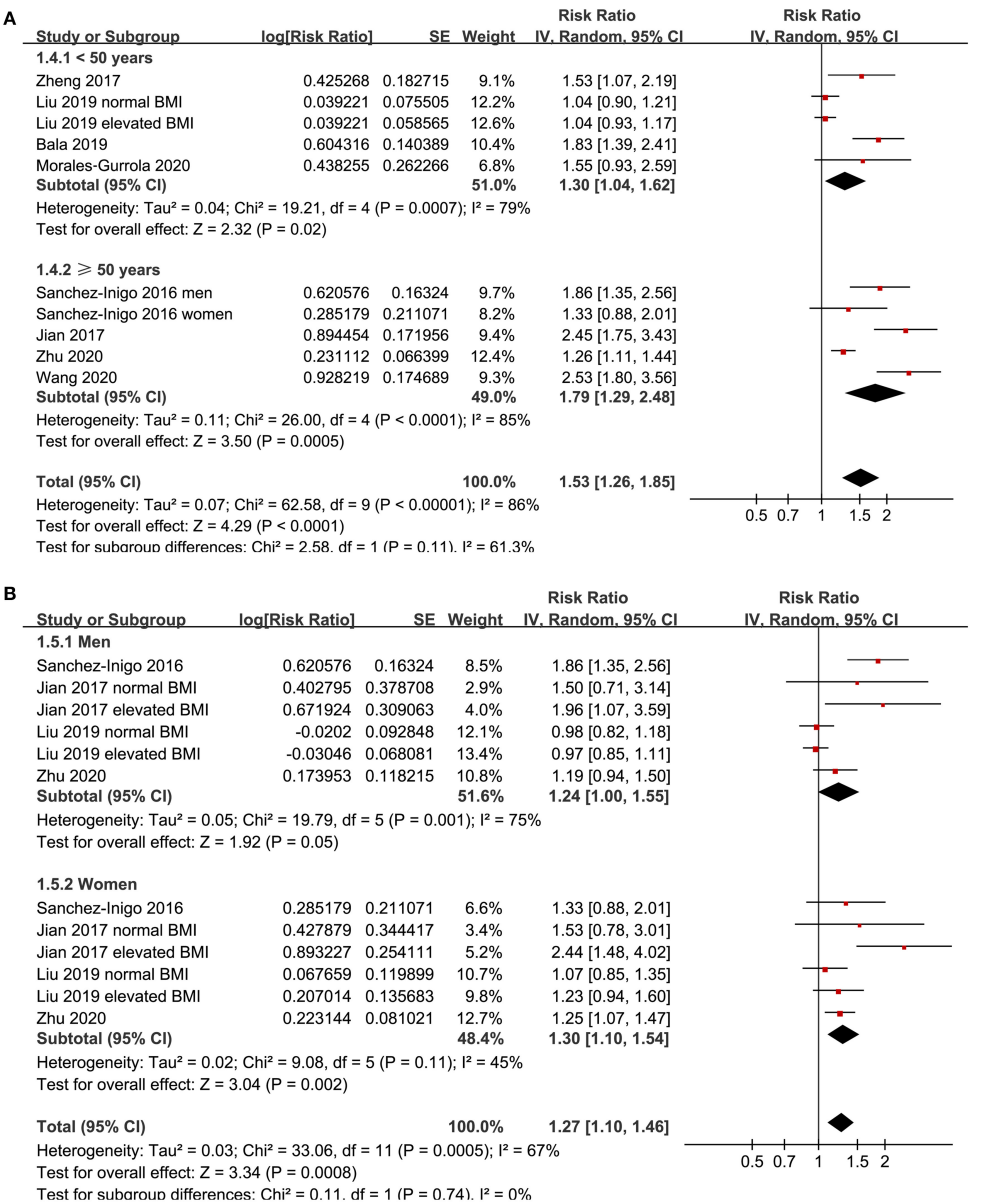
Subgroup analyses for the association between TyG index and hypertension in community-derived adult population; **(A)** subgroup analysis according to the mean age of the population; and **(B)** subgroup analysis according to the gender of the participants.

Eight studies (11–18) evaluated the odds of hypertension in community derived adult population with highest vs. lowest TyG index. Pooled results with a random-effect model showed that participants with the highest TyG index had higher odds hypertension (adjusted RR: 1.53, 95% CI: 1.26–1.85, *I*^2^ = 54%, *P* < 0.001; Figure 2) compared with those with the lowest TyG index. Sensitivity analyses by omitting one study at a time showed similar results (RR: 1.44–1.62, *P* all < 0.001; Table 2). Results of univariate meta-regression analysis are shown in Table 3, which showed that differences in sample size, mean age, male proportion, mean body mass index, and study quality score among the included studies did not have significant influence on the association between TyG index and hypertension (*P* values all > 0.10). These results suggested that differences in these characteristics may not be the major source of heterogeneity. Moreover, subgroup analyses showed consistent association in prospective studies (RR: 1.60, 95% CI: 1.30–1.97, *P* < 0.001) and cross-sectional studies (RR: 1.51, 95% CI: 1.20–1.91, *P* < 0.001; Figure 3A), in Chinese (RR: 1.46, 95% CI: 1.15–1.86, *P* = 0.002) and non-Chinese (RR: 1.70, 95% CI: 1.43–2.03, *P* = 0.001; Figure 3B) population, in participants with mean age <50 years (RR: 1.30, 95% CI: 1.04–1.62, *P* = 0.02) and ≥50 years (RR: 1.79, 95% CI: 1.29–2.48, *P* < 0.001; Figure 4A), and in men (RR: 1.24, 95% CI: 1.00–1.55, *P* = 0.05) and women (RR: 1.30, 95% CI: 1.10–1.54, *P* = 0.002; Figure 4B).

**Table 2:** Results of sensitivity analysis.

**Dataset excluded**	**RR**	**95% CI**	***I^**2**^*%**	***P* for effect**
Sanchez-Inigo et al. (11) men	1.49	1.22–1.83	86	<0.001
Sanchez-Inigo et al. (11) women	1.55	1.26–1.90	87	<0.001
Jian et al. (12)	1.44	1.20–1.74	83	<0.001
Zheng and Mao (13)	1.53	1.24–1.88	87	<0.001
Liu et al. (15) normal BMI	1.62	1.30–2.01	85	<0.001
Liu et al. (15) elevated BMI	1.62	1.30–2.00	83	<0.001
Bala et al. (14)	1.49	1.22–1.83	85	<0.001
Zhu et al. (18)	1.58	1.24–2.01	87	<0.001
Morales-Gurrola et al. (16)	1.53	1.25–1.87	87	<0.001
Wang et al. (17)	1.44	1.20–1.73	83	<0.001

*RR, risk ratio; CI, confidence interval; BMI, body mass index*.

**Table 3:** Impact of study characteristics on the association between TyG index and odds of hypertension: univariate meta-regression analysis.

	**Relative risk of hypertension**		
	**Coefficient**	**95% CI**	***P***
Number of subjects	−0.00004	−0.00012 to 0.00005	0.24
Mean age (years)	0.024	−0.006 to 0.054	0.11
Male (%)	0.0005	−0.098 to 0.099	0.98
Mean BMI (kg/m2)	0.054	−0.074 to 0.183	0.36
Quality score	0.44	−0.08 to 0.96	0.12

*BMI, body mass index; CI, confidence interval*.

### Publication Bias

**Figure 5:**
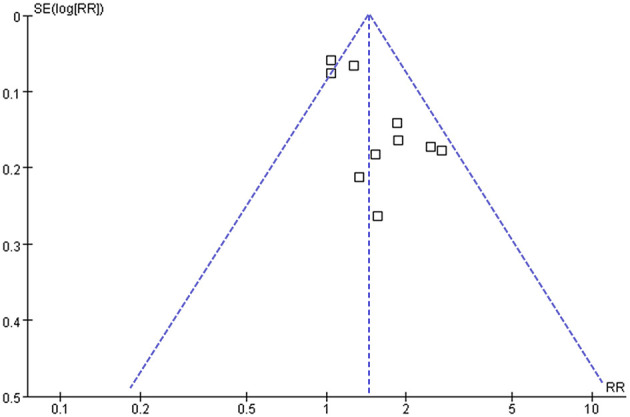
Funnel plots for the publication bias underlying the meta-analysis of the association between TyG index and hypertension in community-derived adult population.

The funnel plots regarding the association between serum TyG index and hypertension are shown in Figure 5. The funnel plots were symmetry on visual inspection, suggesting low risk of publication bias. Egger's regression test and Begg's test also showed consistent results (*P* = 0.28 and 0.19, respectively).

## Discussion

In this meta-analysis of observational studies, we found that compared with those with the lowest category of TyG index, adults with the highest category of TyG index were independently associated with higher odds of hypertension. Besides, consistent results were obtained in subgroup analyses according to the study design, ethnicity, age, and sex of the participants. Taken together, these findings suggested that higher TyG index may be associated with higher odds of hypertension in general adult population. Although these findings should be validated in large-scale prospective cohort studies, results of our study suggest that TyG index, an easily obtained indicator of insulin resistance, could be applied as a predictor of hypertension risk in general adult population. Moreover, the potential pathophysiological mechanisms underlying the association between TyG index and hypertension deserve further investigation.

To the best of our knowledge, our study is the first meta-analysis which evaluated the association between TyG index and hypertension in community-derived general population. The strengths of our meta-analysis include the following: First, only studies with multivariate analysis were included, which therefore could provide an independent association between TyG index and hypertension. In addition, sensitivity analysis was performed to evaluate the stability of the results, which confirmed that the association between TyG index and hypertension was not primarily driven by either of the included studies. Finally, multiple predefined subgroup analyses were applied to evaluate the robustness of the findings, which showed that the association between TyG index and hypertension was not affected by the differences in study design, ethnicity, age, or gender of the participants. Our findings may reflect the pathogenetic association between insulin resistance and hypertension. Previous studies showed that insulin resistance is characterized of low-degree systematic inflammation, which may cause endothelial dysfunction, one of the initial pathogenic processes underlying arterial hypertension (26, 27). Besides, insulin resistance may also affect renal sodium metabolism (28), increase the activity of the sympathetic nerve system (29), and modulation the secretion of vasoactive substances (30), all of which have been implicated in the pathogenesis of hypertension.

An important clinical implication of our study is that TyG index may be applied as an indicator to reflect the risk of hypertension in general adult population (31). Compared with the hyperinsulinemic–euglycemic clamp test, TyG index could be easily obtained because measuring triglyceride and glucose is inexpensive and routine. The TyG index had high sensitivity (96.5%) and specificity (85.0%) for detection of insulin resistance as compared with the hyperinsulinemic–euglycemic clamp test, as evidenced by a previous study in Mexico (32). Besides, TyG index showed better performance than homeostatic model assessment for measuring insulin resistance (33). Large-scale studies evaluating the temporal relationship between baseline TyG index and subsequent risk of hypertension in general population are needed to validate the findings of the meta-analysis.

Some limitations should be noticed when the results of the meta-analysis are interpreted. First, the datasets available for the meta-analysis were limited, and the results for the subgroup analyses should be interpreted with caution due to the small number of datasets and participants included. Second, although only studies with multivariate analyses were included, we could not exclude the possibility of unadjusted residual factors that may confound the association between TyG index and hypertension, such as dietary factor of the included populations and the concurrent medications used. Moreover, significant heterogeneity was observed among the included studies. However, results of univariate meta-regression analysis showed that differences in sample size, mean age, male proportion, mean BMI, and study quality score among the included studies did not have significant influence on the association between TyG index and hypertension (*P* values all > 0.10), suggesting that differences in these characteristics may not be the major source of heterogeneity. Besides, our predefined subgroup analyses also did not support that the varying results of the included studies could be explained by differences in study design, patient ethnicity, age, or sex. Currently, we are unable to determine the possible reasons of inconsistent results among the included studies. However, difference in some other related factors (comorbidities, concurrent medications, dietary factors, or cut-off values for TyG index) may be responsible for the inconsistent results among the studies on the association between triglyceride-glucose index and hypertension. Large-scale prospective cohort studies are warranted to validate our findings and determine the potential influences of patient characteristics on the association between TyG index and hypertension risk. Besides, combining the results of studies with cross-sectional and cohort design could also contribute to the heterogeneity, although the results of subgroup analysis according to the study design showed no statistical significance. In addition, it remains unknown whether the association between TyG index and hypertension is linear because we only included categorized data. In fact, to the best of our knowledge, only one study showed that TyG index as a continuous variable remained to be associated with hypertension in elderly individuals from China (18). Moreover, although both Egger's regression test and Begg's test suggested low risk of publication bias, these results should be interpreted with caution since only 10 datasets were included in the meta-analysis. Finally, a causative association between higher TyG index and hypertension could not be derived from this study because it is a meta-analysis of observational studies.

In conclusion, current evidence from observational studies suggests that higher TyG index may be associated with higher odds of hypertension in general adult population. Large-scale prospective cohort studies are needed to validate these findings, and further studies are needed to elucidate the potential pathophysiological mechanisms underlying the association between TyG index and hypertension.

## Data Availability Statement

The original contributions presented in the study are included in the article/supplementary material, further inquiries can be directed to the corresponding authors.

## Author Contributions

YW and XJ designed the study. YW and WY performed the literature search, data extraction, quality evaluation, and wrote the manuscript. All authors performed the statistical analyses, reviewed and revised the manuscript, and approved the manuscript for submission.

## Conflict of Interest

The authors declare that the research was conducted in the absence of any commercial or financial relationships that could be construed as a potential conflict of interest.
